# Don’t quote me: reverse identification of research participants in social media studies

**DOI:** 10.1038/s41746-018-0036-2

**Published:** 2018-08-02

**Authors:** John W. Ayers, Theodore L. Caputi, Camille Nebeker, Mark Dredze

**Affiliations:** 1 Division of Infectious Disease and Global Public Health, University of California San Diego, School of Medicine, La Jolla, CA USA; 2 School of Public Health, College of Medicine and Health, University College Cork, Cork, Ireland; 3 Department of Family Medicine and Public Health, University of California San Diego, School of Medicine, La Jolla, CA USA; 4 Department of Computer Science, Johns Hopkins University, Baltimore, MD USA

**Keywords:** Epidemiology, Translational research

## Abstract

We investigated if participants in social media surveillance studies could be reverse identified by reviewing all articles published on PubMed in 2015 or 2016 with the words “Twitter” and either “read,” “coded,” or “content” in the title or abstract. Seventy-two percent (95% CI: 63–80) of articles quoted at least one participant’s tweet and searching for the quoted content led to the participant 84% (95% CI: 74–91) of the time. Twenty-one percent (95% CI: 13–29) of articles disclosed a participant’s Twitter username thereby making the participant immediately identifiable. Only one article reported obtaining consent to disclose identifying information and institutional review board (IRB) involvement was mentioned in only 40% (95% CI: 31–50) of articles, of which 17% (95% CI: 10–25) received IRB-approval and 23% (95% CI:16–32) were deemed exempt. Biomedical publications are routinely including identifiable information by quoting tweets or revealing usernames which, in turn, violates ICMJE ethical standards governing scientific ethics, even though said content is scientifically unnecessary. We propose that authors convey aggregate findings without revealing participants’ identities, editors refuse to publish reports that reveal a participant’s identity, and IRBs attend to these privacy issues when reviewing studies involving social media data. These strategies together will ensure participants are protected going forward.

## Introduction

Social media surveillance is increasingly used to track public health trends because it can reveal what the public is thinking or doing based on the content of their public posts.^1,2^ Potential ethical issues exist in the use of such data.^3–8^ One overlooked issue is the inclusion of direct quotes or usernames of social media users in academic publications. When preserved this way, the quoted material can potentially be linked back to the originating account and inferentially the account owner. Given the resulting privacy implications, we investigated how common these practices are in the medical literature and whether participants could be reverse identified.

## Results

Two-hundred-eleven publications matched our search criteria, of which 115 focused on population health or surveillance. Three publications could not be accessed because the link was broken or we could not eclipse the journal’s paywall, leaving a corpus of 112 papers for analysis.

Eighty-one (72%; 95% CI: 63–80) articles quoted at least one tweet. In 68 (61%; 95% CI: 51–70) of these, we identified at least one quoted account holder, representing 84% (95% CI: 74–91) of articles with quoted tweets. Twenty-three (21%; 95% CI: 13–29) disclosed a participant’s Twitter username and in all cases the participant was reverse identified.

Only one study reported explicitly obtaining consent to disclose identifying information. IRB or ethical review was mentioned in 45 (40%; 95% CI: 31–50) studies, of which 19 (17%, 95% CI: 10–25) received IRB-approval, and 26 (23%, 95% CI:16–32) were deemed exempt.

## Discussion

Studies mining Twitter frequently included content, such as quotes or usernames, that could be traced back to the original poster; nearly all without consent and most occurring outside IRB review.

While Twitter’s data sharing policy permits quoting social media posts or disclosing usernames, in the academic literature this is a violation of the *International Committee of Medical Journal Editors* (ICMJE) ethics standards. The ICMJE states “identifying information…should not be published in written descriptions, photographs, or pedigrees unless the information is essential for scientific purposes and the [participant] gives written informed consent for publication” after reviewing the manuscript prior to publication.^9^ Disregarding these guidelines, authors and editors are authorizing the exposure of potentially identifiable information that could be linked to medical diagnoses, drug use, or other sensitive topics.

It is imperative that we protect participant privacy even in social media studies. First, privacy settings are set by the account owner who may post sensitive information and then later delete or make their post private. There are documented cases of people compromising their job, college admission, or relationships when their postings were rebroadcast on other media channels.^10^ Publication in the biomedical literature is permanent and removes control from the poster. Second, revealing the identity of a participant adds no scientific value given all the studies we reviewed aimed to make population (not individual) inferences. Sharing a username or quoting their content is immaterial to the aims of these studies.

Our study was limited to publications using Twitter and it is unclear whether works using other social media data also expose participants. This paper is designed to be an exploratory rather than systematic review, and so there is a chance we missed articles in our search strategy that may have fit our inclusion criteria (however, the 115 articles we analyzed were sufficient to capture the scale of the problem). Regarding IRB involvement, it is possible that authors obtained appropriate IRB review but did not explicitly describe the details in their manuscript.

Researchers must apply the same protocols to protect social media users as they do for any other study participant. We propose that authors convey aggregate findings without revealing participants’ identities, editors refuse to publish reports that potentially reveal a participant’s identity unless it is scientifically necessary and informed consent is obtained, and IRBs attend to these privacy issues when reviewing studies involving social media data. These strategies together will ensure the identity of participants are protected going forward.

## Methods

We searched PubMed for all articles published in 2015 or 2016 that included the words “Twitter” and “read,” “coded,” or “content” in the title or abstract. Researchers typically describe observational analyses as “content analyses” or “coded Twitter postings,” meaning our search should return articles focused on mining Twitter data. Articles primarily about population health were then selected for inclusion. Excluded articles were surveys using Twitter as a sampling frame, experimental studies testing marketing strategies on Twitter, and editorials.

T.L.C. and J.W.A. independently assessed whether articles: (a) quoted a tweet, (b) included a participant’s twitter username, (c) if any disclosed participant was reverse identifiable, (d) if consent for revealing a participant was obtained, (e) if institutional review board (IRB)-review was mentioned, and (f) if IRB-approval/exemption was given. The authors discussed coding discrepancies until reaching agreement on all labels. Frequencies for each outcome along with binomial confidence intervals were computed using R Ver. 3.4.1. Given our data was the published literature, we did not seek IRB review.

### Data availability statement

The data used in the study were the studies resulting from a PubMed search. A listing of articles and our final coding of the studies are available upon request.

### Disclaimer

Dr. Ayers and Mr. Caputi had full access to all the data in the study and take responsibility for the integrity of the data and the accuracy of the data analysis.
